# Supplementary material for: Identification of Freezing-Responsive microRNAs and Their Targets in Chinese Jujube by Small RNA and Degradome Sequencing
Source: Int J Mol Sci. 2026 Sep 4;27(17):7912. doi: 10.3390/ijms27177912 (PMC13566890; doi:10.3390/ijms27177912)
Supplement: Supplementary file 1 [file ijms-27-07912-s001.zip › Supplementary Material S2/novel_miR_96.pdf]

Provisional ID	:	novel_miR_96
Score total	:	0.4
Score for star read(s)	:	-1.3
Score for read counts	:	0
Score for mfe	:	0.1
Score for randfold	:	1.6
Score for cons. seed	:	
Total read count	:	79
Mature read count	:	79
Loop read count	:	0
Star read count	:	0

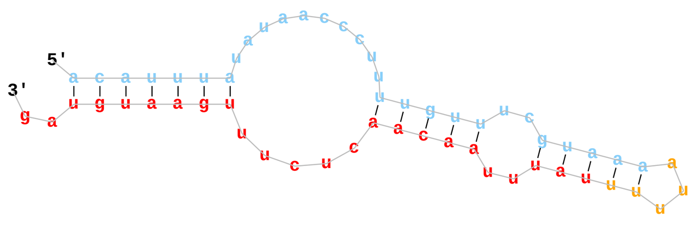

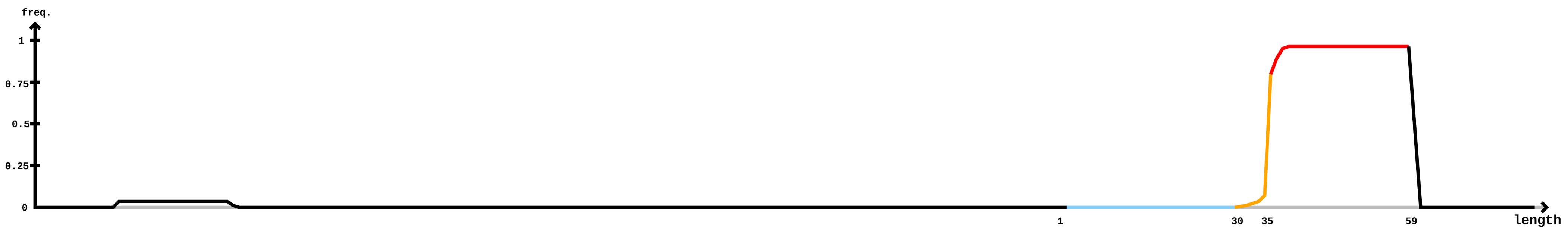

Star Mature

```
5'- uucuuuuuuaaguugguuaaggauuaaaaauuucauuuuucgauuaucuuuuggcuuucagauuuuacuugaguagaccuuuguuaugguaaaguauguucuggaauacaauugaagauuguugaauaugauuaaucauaauauuaauaauagaugaauaccucaaaucuacauuuauauaacccuuuuguuucguaaaauuuuuauuuaacaacucuuugaauguagagacuggugguuucaauagcau -3'   exp
    ...........((((((....((((((.....((((..((((((.((((((((..(((((((...(((((.....(((........))).)))))...)))))))..))...)))))).)))))).)))))))))).....))))))......((((.((((((..((((((((((.........(((((..(((((...)))))..))))).....))))))))))...))).)))))))......   reads   mm   sample
    ............uugguuaUggauuaaaaau...................................................................................................................................................................................................................   2   1   JCK-3
    ............uugguuaUggauuaaaaauu..................................................................................................................................................................................................................   1   1   LT-1
    ........................................................................................................................................................................................................uuuuuauuuaacaacucuuugaauguag......................   1   0   LT-2
    .........................................................................................................................................................................................................uuuuauuuaacaacucuuugaauguag......................   1   0   LT-2
    ..........................................................................................................................................................................................................uuuauuuaacaacucuuugaauguag......................   1   0   LT-1
    ...........................................................................................................................................................................................................uuauuuaacaacucuuugaauguag......................   1   0   LT-1
    ...........................................................................................................................................................................................................uuauuuaacaacucuuugaauguag......................   1   0   JCK-2
    ...........................................................................................................................................................................................................uuauuuaacaacucuuugaauguag......................   1   0   LT-2
    ............................................................................................................................................................................................................uauuuaacaacucuuugaauguag......................   4   0   LT-1
    ............................................................................................................................................................................................................uauuuaacaacucuuugaauguag......................   16   0   LCK-2
    ............................................................................................................................................................................................................uauuuaacaacucuuugaauguag......................   25   0   LCK-1
    ............................................................................................................................................................................................................uauuuaacaacucuuugaauguag......................   16   0   LCK-3
    .............................................................................................................................................................................................................auuuaacaacucuuugaauguag......................   3   0   LCK-3
    .............................................................................................................................................................................................................auuuaacaacucuuugaauguag......................   4   0   LT-2
    .............................................................................................................................................................................................................auuuaacaacucuuugaauguag......................   1   0   LT-1
    ..............................................................................................................................................................................................................uuuaacaacucuuugaauguag......................   5   0   LCK-3
    ...............................................................................................................................................................................................................uuaacaacucuuugaauguag......................   1   0   LT-1
```
